# Navigating Decisional Capacity Assessments for People with Dementia: Social Workers’ Strategies and Challenges at the Intersection of Cognitive Impairment and Social Inequality

**DOI:** 10.1002/alz70858_097224

**Published:** 2025-12-24

**Authors:** Xueping Ma, Sussman Tamara, Sarah Donnelly, Shari Brotman

**Affiliations:** ^1^ McGill University, Montreal‐west, QC, Canada; ^2^ McGill University, Montreal, ON, Canada; ^3^ University college Dublin, Belfield, Dublin, Ireland; ^4^ McGill University, Montreal, QC, Canada

## Abstract

**Background:**

Legislation across the globe has called on social workers and other health professionals to support the rights of persons with dementia to remain as autonomous as possible in all aspects of their lives. Yet, protecting rights can become complex when professionals are also called on to assess for decisional capacity.

**Objective:**

This study explores social workers’ experiences conducting psychosocial capacity assessments with persons with dementia at long‐term and home care settings, and the strategies they use to support decisional inclusion, autonomy, and empowerment.

**Methods:**

Guided by the principles of constructivist grounded theory (CGT), five in‐depth interviews were conducted with social workers, 4 worked at LTC and 1 worked at homecare, and they have been called on to participate in at least one capacity assessment in the last five years in Montreal Quebec Canada. All interviews were transcribed and analyzed thematically.

**Results:**

Our findings suggest that social inequities such as ageism, ableism, and racism, the absence of family or community support and scarcity of resources can work together to erode the persons with dementia's sense of autonomy. Social workers emphasized that building trust, engaging families and communities, and addressing broader social issues are paramount to help foster inclusion and empower individuals within decision‐making contexts. However, attending to these critical issues can be challenging without the necessary organizational support.

**Conclusion and Implications:**

Supporting persons with dementia during capacity assessments is a complex process that demands social workers to navigate a web of social, organizational, and interpersonal challenges. While relational strategies—like strengthening social connections and empowering persons with dementia—offer promising ways to address these difficulties, tight organizational budgets and competing priorities can create barriers to this work. Ultimately, social workers’ success in promoting autonomy and inclusion depends not only on their individual efforts, but also on the systemic conditions that shape their work.

